# Supplementary material for: Fisetin may protect early porcine embryos from oxidative stress by down-regulating GRP78 levels
Source: PeerJ. 2025 Mar 28;13:e19198. doi: 10.7717/peerj.19198 (PMC11956767; doi:10.7717/peerj.19198)
Supplement: Supplemental Information 13 [file peerj-13-19198-s013.pptx]

## Slide 1
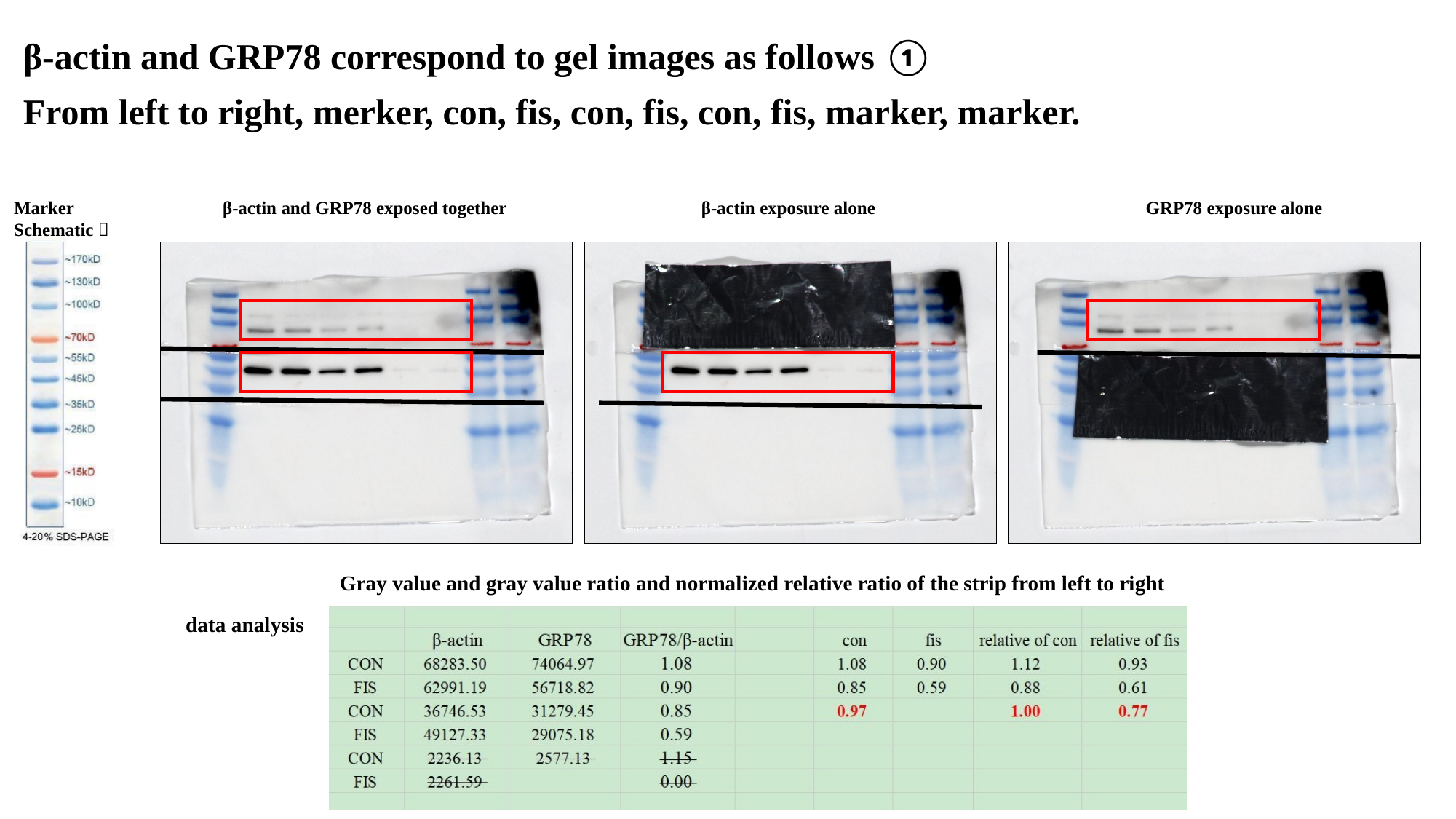

β-actin and GRP78 correspond to gel images as follows ①
From left to right, merker, con, fis, con, fis, con, fis, marker, marker.
GRP78 exposure alone
Marker Schematic：
β-actin and GRP78 exposed together
β-actin exposure alone
Gray value and gray value ratio and normalized relative ratio of the strip from left to right
data analysis

## Slide 2
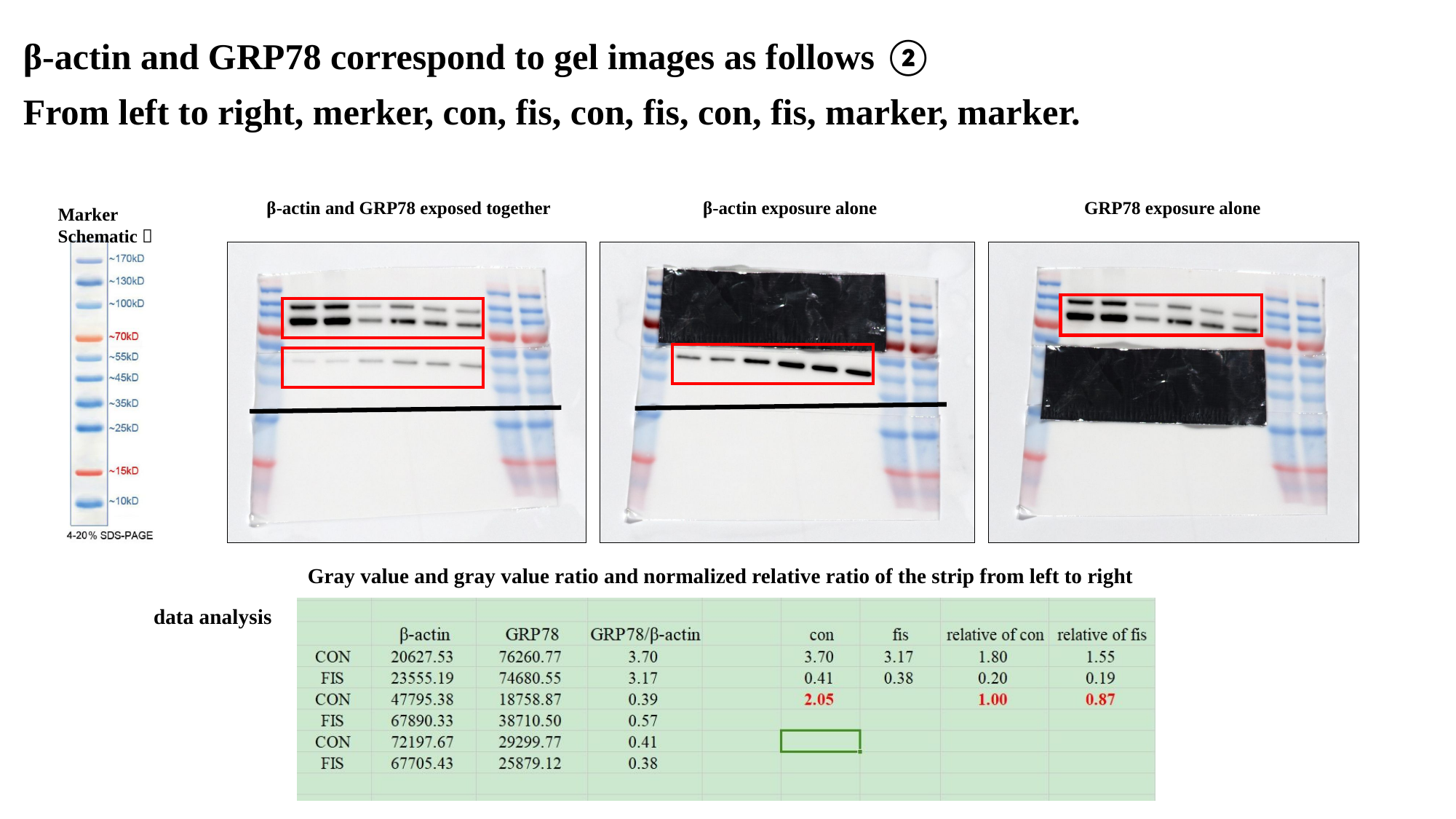

β-actin and GRP78 correspond to gel images as follows ②
From left to right, merker, con, fis, con, fis, con, fis, marker, marker.
GRP78 exposure alone
β-actin and GRP78 exposed together
β-actin exposure alone
Marker Schematic：
Gray value and gray value ratio and normalized relative ratio of the strip from left to right
data analysis

## Slide 3
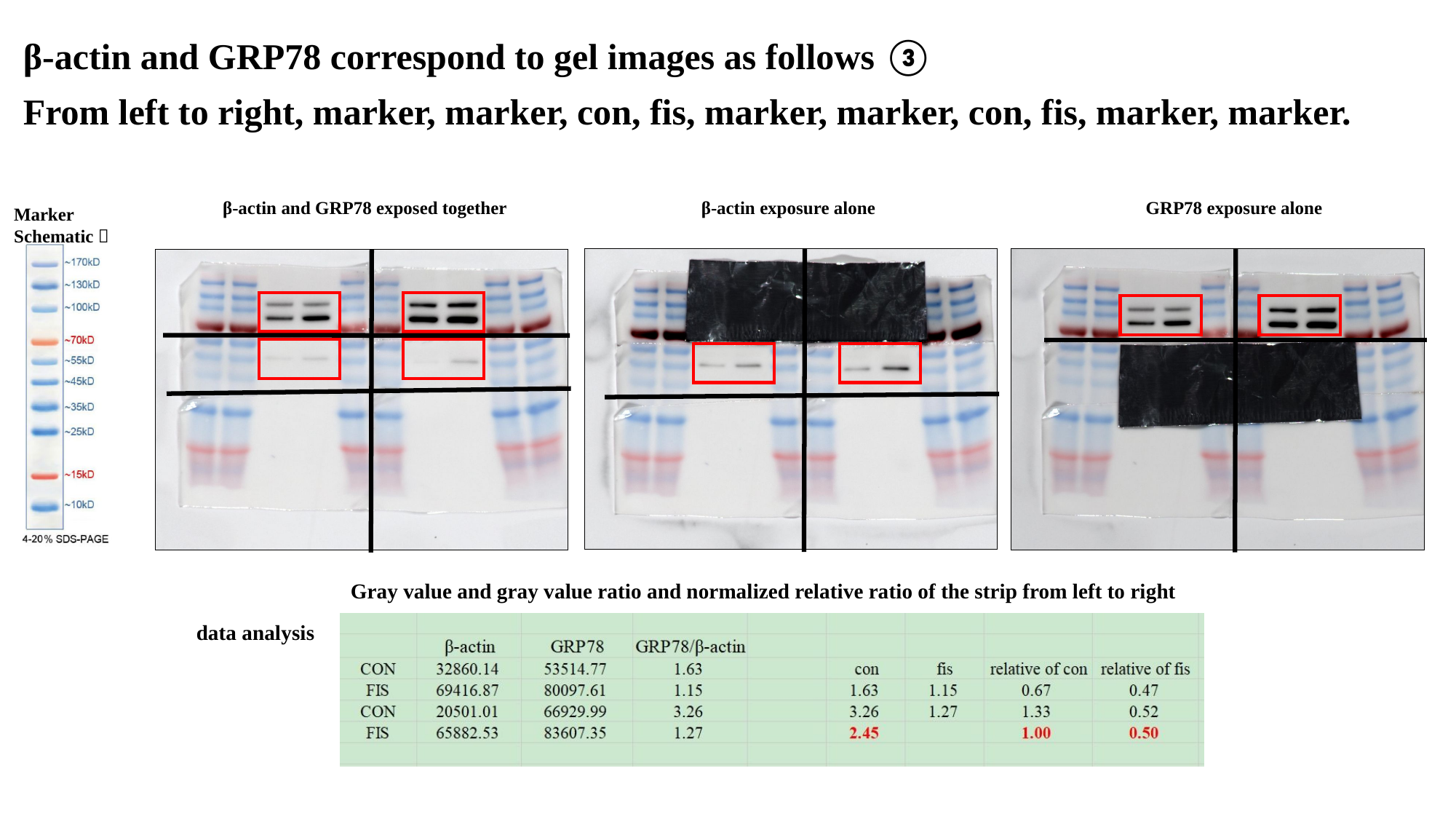

β-actin and GRP78 correspond to gel images as follows ③
From left to right, marker, marker, con, fis, marker, marker, con, fis, marker, marker.
GRP78 exposure alone
β-actin and GRP78 exposed together
β-actin exposure alone
Marker Schematic：
Gray value and gray value ratio and normalized relative ratio of the strip from left to right
data analysis
